# Antimicrobial Use in On-Farm Hatching Systems vs. Traditional Hatching Systems: A Case Study

**DOI:** 10.3390/ani13203270

**Published:** 2023-10-19

**Authors:** Julia G. Jerab, Ilias Chantziaras, Tommy Van Limbergen, Johan Van Erum, Filip Boel, Erik Hoeven, Jeroen Dewulf

**Affiliations:** 1 Veterinary Epidemiology Unit, Faculty of Veterinary Medicine, Ghent University, Salisburylaan 133, 9820 Merelbeke, Belgium; ilias.chantziaras@ugent.be (I.C.); jeroen.dewulf@ugent.be (J.D.); 2 Pehestat BV, Dwarsstraat 5, 3560 Lummen, Belgium; tommy.vanlimbergen@anitom.be (T.V.L.); johan.van.erum@galluvet.be (J.V.E.); 3 Belgabroed, Steenweg op Hoogstraten 141, 2330 Merksplas, Belgium; f.boel@belgabroed.be; 4 HFHC NV, Nerm 94, 3320 Hoegaarden, Belgium; info@nestborn.eu

**Keywords:** antimicrobial, broiler, on-farm hatching

## Abstract

**Simple Summary:**

Antimicrobial resistance is a worldwide problem that requires innovation on various fronts. In a traditional broiler hatchery system, hatched chicks remain in the incubator for between 24 and 48 h, after which the chicks are transported to poultry farms. During this period (up to 72 h), the chicks have no access to feed or water and are exposed to dust and pathogens. Research shows that feed and water deprivation has a negative impact on the development of the gastrointestinal and immune system of broilers. In an on-farm hatching system (NestBorn), eggs are transported to the broiler farm on day 18 of the incubation period, where the chicks have direct access to feed and water after hatching. This would result in better animal welfare, a healthier gastrointestinal system, and less antimicrobial use, among other things. This study compared antimicrobial use in 227 on-farm and 2244 traditionally hatched flocks. It found that on-farm hatched broilers had a lower antimicrobial treatment incidence and had more antimicrobial free flocks. The use of on-farm hatching also resulted in a 5.6 times lower probability of antimicrobial use. These results indicate that on-farm hatching may contribute substantially to the decrease in antimicrobial use in broilers and thereby could play an important role in the future of a more sustainable and ethical broiler production.

**Abstract:**

On-farm hatching is a relatively new method in the broiler industry, in which fertilized broiler eggs are transported to the farms at the stage of 17–19 days of incubation. Once hatched, the broiler chicks have direct access to feed and water. Previous studies have shown on-farm hatching to increase animal welfare and intestinal development. However, no studies have yet aimed to quantify and compare the antimicrobial use in on-farm hatched flocks with that of traditionally hatched flocks. In this study, information on antimicrobial use (AMU) was collected from 211 Belgian conventional broiler farms, including data from 2244 traditionally hatched flocks and 227 on-farm (NestBorn) hatched flocks. On-farm hatched flocks had significantly (*p* < 0.001) more antimicrobial-free flocks (*n* = 109, 48.01%) compared to traditional flocks (*n* = 271, 12.08%) and a 44% lower (*p* < 0.01) treatment incidence (TI) at flock level (TI 8.40 vs. TI 15.13). Overall, the farms using traditional hatching had 5.6 times (95% CI 3.6–8.7) higher odds to use antimicrobials than the farms using on-farm hatching. Treated on-farm hatched flocks received three times less lincomycin-spectinomycin (linco-spectin) and less (routine) treatments at the start of the production round. However, both traditional and on-farm flocks experienced outbreaks later in the production round. These results show that on-farm hatching can contribute to the reduction in antimicrobial use in conventional broiler production.

## 1. Introduction

Since their discovery in the 1920′s, antimicrobials have been widely used in animals, especially in the food animal industry [1]. Worldwide, 60% of the antimicrobials that are produced are used in animal production [2]. Broiler production is at the forefront of the increased global demand for animal protein, with the highest absolute growth rate and projected growth rate of all meat sources [3]. However, in broiler production, antimicrobials often play an important role in the management of animal health [4]. Yet, antimicrobial resistance (AMR) is one health threat, with the use of antimicrobials being the main driver for resistance selection [3,5]. Acknowledging the latter, the European Union (EU) has taken several initiatives to address the prudent use of antimicrobials in animals [6,7]. In the same spirit, the recent implementation of the EU regulation 2019/6 aims to further reduce antimicrobial use within the veterinary sector, thereby increasing the pressure on the livestock industry to use antimicrobials more prudently [8]. It is evident that there is a global need for alternatives for antimicrobial use.

In the current (traditional) hatching systems, the norm is that chicks remain in the hatchers until all chicks have hatched during the foreseen ‘hatching window’, ranging between 24–48 h. After pulling from the hatchers, the chicks undergo handlings such as separation, grading, counting, vaccination, and packaging and are transported to the broiler farms [9]. Throughout this period, the chicks usually have no access to feed and water, resulting in a total period of 48–72 h of feed and water deprivation [10]. Studies indicate that the absence of feed and water post-hatching have a negative effect on the performance [11] and the morphological development of the intestinal mucosa [12,13], as well as an adverse effect on the immunological development in chicks [9,14]. Other stressors that impact the health and welfare of the chicks in traditional hatching systems are handling, cross contamination, high exposure to dust and pathogens [15], and transport [16].

In on-farm hatching systems, eggs are brought to the broiler farm at day 18 of incubation (range is 17–19 days), where the chicks will have direct and uninterrupted access to feed and water after hatching. Commercial on-farm hatching systems claim to have a variety of benefits in comparison to traditional hatching systems. 

De Jong et al. [17] found that on-farm hatching reduced overall mortality, thus improving broiler welfare. In the same study, the live weight of chicks raised in on-farm hatching systems was significantly higher than that of chicks raised in traditional hatching systems. The latter was also found in a study performed by Jessen et al. [18]. Dibner et al. [19] found that early feeding had positive effects on the development of the immune system of chicks, as well as on the bird’s performance in fighting off a disease challenge. Regarding chick welfare, studies have shown positive results with improved welfare of chicks in early feeding and on-farm hatching methods [20]. Two scientific opinions of the European Food Safety Authority’s (EFSA) state that transporting fertilized eggs in the context of on-farm hatching is the only manner to avoid welfare consequences during transport and on broiler farm [21,22].

While such studies provide scientific support on certain positive claims made by commercial on-farm hatching companies, data regarding a lower antimicrobial use in on-farm hatching systems, compared to traditional hatching systems, are limited, and claims are mainly made based on empirical observations. Therefore, this study aims to collect, quantify, and compare antimicrobial use in flocks that were hatched using traditional hatching systems and with those hatched using on-farm hatching.

## 2. Materials and Methods

In this cohort study, the antimicrobial use (AMU) data were collected by PEHESTAT (Lummen, Belgium) at 211 Belgian broiler farms. PEHESTAT is a Belgian company linked to specialized poultry veterinary companies with the purpose of collecting and managing veterinary-based data. Of these 211 farms, 204 farms (415 houses) applied traditional hatching methods, whereas 28 farms (54 houses) applied on-farm hatching methods. Of the 204 farms that applied traditional hatching at the start of the period of data collection, 21 farms (36 barns) moved to on-farm hatching during this period (1 year). In total, the AMU data in 2244 traditionally hatched flocks and 227 on-farm hatched flocks was collected. All farms had conventional broiler production, with no slow growing breeds and an average rearing period of 42 days. All broilers data included in the dataset originated from two Belgian hatcheries that were both part of the same integration with common health and management amongst all broiler breeders.

NestBorn (NB, Hoegaarden, Belgium) is a Belgian company that provides a commercial on-farm hatching system. In farms using the NestBorn system, the broiler houses are first prepared by disinfecting and pre-heating to a maximum of 28° Celsius floor temperature. The pre-incubated eggs are transported in pre-heated and disinfected trucks from the hatchery to the farm, where they are then placed onto the litter bed using the NestBorn egg placing machine. Typically, the completion of the hatching process takes three days, during which the eggs can be monitored via the NestBorn Monitoring Platform, which measures the eggshell temperatures and climate conditions of the poultry house. Hatched chicks then have direct access to feed and water and are free from chick handling and transport [23].

### 2.1. Flock Size and Husbandry Conditions

The average flock size at the start of the production round was 30,266 broilers for on-farm hatched flocks and 30,287 for traditionally hatched flocks. Both traditional and on-farm flocks aimed to have a density of 42 kg/m^2^, which was equivalent to 21–22 chicks per m^2^. As 100% of the eggs were not placed in the stable for on-farm hatching, a factor of 3.5% was added to the amount of delivered eggs, to ensure the same density as when traditionally hatched chicks were used as much as possible. All flocks, regardless of hatching method, were thinned once (25% of chicks at a weight of +/−2 kg), and the remaining 75% were slaughtered at day 40–42, with a final weight of around 2.7 kg. In on-farm hatched flocks, vaccination occurred on the ‘day of set up’, which was the day that the chicks would have been transported to the farm, had they been traditionally hatched, and coincided with day 0. Thus, the moment of vaccination did not differ between the two groups.

### 2.2. Study Sample and Data Collection

Between 2 January 2020 and 31 December 2020, the AMU of all broiler farms that received eggs or day-old chicks from the two hatcheries involved in this study were collected via an automated data-collection system. Data contained the main characteristics of farms, houses, flocks, and AMU. The farm data included number of houses per farm and total capacity of broilers, whereas the house data included capacity of broilers in the house. The flock data included date of placement, house identification, hatchery identification, and type of production, i.e., traditional hatching or on-farm hatching. AMU data included molecule, antimicrobial class, dosage, indication, and age at treatment.

### 2.3. AMU Quantification

In order to quantify AMU in a standardized manner, treatment incidence (TI), as described by Persoons et al. [24], was used. Using this method, the numerator of the TI equalled the total quantity of active substance (AS) purchased, which was calculated by multiplying the number of packages purchased with the package size and the concentration of AS in the product. The denominator of the TI equalled the Defined Daily Dose (DDDvet). Thus, the TI represented the number of DDDvet administered per 100 animal days at risk (AAR), using the following formula:

$$\frac{total\;amount\;of\;AS\;purchased}{DDDvet\;\left(mg/kg/day\right)\;\times \;no.of\;days\;at\;risk\;\times \;kg\;of\;AAR}\;\times \;100\;AAR$$

The DDDvet was defined as the assumed average dose of a drug for its main indication per day per kg of broiler [25]. For the DDDvet values, those defined by the European Surveillance of Veterinary Antimicrobial Consumption (EVSAC) were used, as most of the antimicrobials purchased were registered for broilers. DDDvet values were generated from the summary of product characteristics (SPC) if combination products were not on the ESVAC list, and the dose of one or both ASs was significantly different from the dose of the single AS products.

In order to determine the ‘kg of animal at risk’, a growth curve for standard broiler breeds was used [26], as no slow growing breeds were used. The ‘number of days at risk’ was set at 42 days, which was the length of conventional broiler production in Belgium.

### 2.4. Quantitative Analysis

All statistical analyses were performed using IBM SPSS version 27^®^ (Armonk, NY, USA). Descriptive information regarding the various parameters included in this study was calculated focusing on the TI levels of the on-farm and traditional flocks. We did not use parametric tests to evaluate the exact levels of the total TI or the TI per antimicrobial class since not all assumptions of using such statistics were met. Instead, non-parametric (Mann–Whitney) tests were performed to investigate differences between the two hatching systems. Also, a binary parameter was constructed to indicate the use or not of antimicrobial within a production round (flock). This parameter was selected as the dependent variable of a generalized estimating equations model with the farm included as subject and barn as the subject effects, the production round as the within-subject effect, and the type of hatching as fixed factor. Then, in a stepwise forward process, additional parameters were added to the model (one at the time). To wit, the flock size and the origin of the broilers (breeder farm) were inserted and tested for statistical significance, so as to be kept in the final model. The correlation structure matrix of the model was autoregressive. For all the above, a *p*-value of ≤0.05 was considered as statistically significant.

### 2.5. Qualitative Analysis

The treatment incidence of each antimicrobial class in their respective hatching system was also classified according to formulary created and distributed by the Knowledge Centre on Antibiotic Use and Resistance in Animals in Belgium [27]. The formulary classifies antimicrobials into three categories based on their importance for public and animal health, according to the lists published by the World Health Organization (WHO) and the World Organization for Animal Health (WOAH). The categories were distinguished by color, with antimicrobials in category yellow having the least specifications and regulations for use, followed by orange and antimicrobials in category red only being allowed for use as a last resort and after diagnostic and sensitivity testing. The indications for AMU, which were included in the sampled farm records, were also recategorized into relevant larger categories (see Table 1).

## 3. Results

### 3.1. AMU at Flock Level

Antimicrobial treatment was administered in 2091 flocks (84.62%) of the 2471 total production flocks included in the dataset, with a remaining 380 antimicrobial-free production flocks. The 2091 treated flocks consisted of 1973 traditionally hatched flocks and 118 on-farm hatched flocks, leaving 271 (12.07%) non-treated traditionally hatched flocks and 109 (48.01%) non-treated on-farm hatched flocks. Farms using traditional hatching systems had 5.6 (95% CI: 3.6–8.8) times higher odds to use antimicrobials than farms using on-farm hatching systems.

When the treatment incidence in all 2471 followed production rounds, regardless of whether or not they received antimicrobials, was compared on basis of the hatching system, on-farm hatched flocks had a 44% lower (*p* < 0.01) treatment incidence (TI 8.40, CI [6.31–10.49]) compared to traditionally hatched flocks (TI 15.11, CI [14.36–15.85]). Breeder farms (*p* = 0.19) and flock size (*p* = 0.83) were also taken into account; however, both did not have a significant impact on the AMU.

When only the treated flocks are considered, on-farm hatching flocks had a slightly lower (*p* < 0.01) median AMU (TI 11.69, CI [8.77–15.12]) compared to traditionally hatched flocks (TI 13.14, CI [12.60–13.71]). In the treated traditional production rounds, 75% of the total treatment incidence took place within the first week, compared to 51% in treated on-farm hatching rounds. Furthermore, in traditionally hatched production rounds, 37% of all antimicrobial administration took place within the first two days of the production round, more than double compared to on-farm hatched flocks (16%).

**Figure 1:**
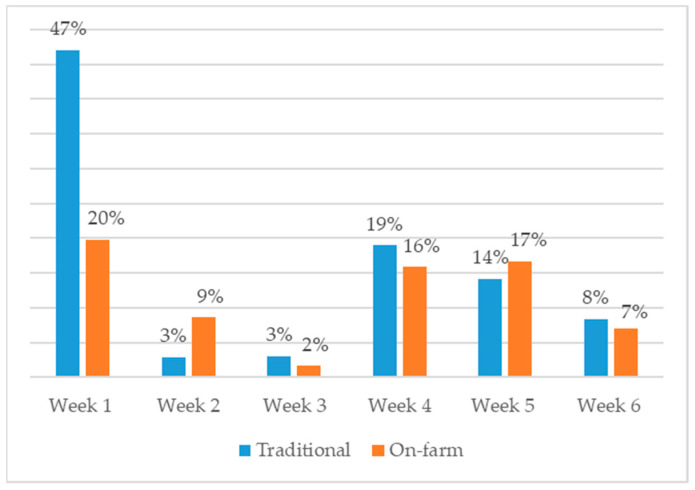
Percentage of traditional (*n* = 2244) and on-farm (*n* = 227) flocks receiving antimicrobial treatments per week.

When comparing the percentage of flocks that received a diagnosis resulting in AMU per week, AMU occurred in 94.62% of the weeks for traditionally hatched flocks, whereas AMU took place in 69.64% of the weeks in on-farm hatched flocks. Both on-farm and traditional flocks experienced an increase in AMU during week four, with 19% and 16% of all flocks (treated and untreated) receiving treatment, respectively (see Figure 1).

Both in traditional production and on farm hatching, lincomycin-spectinomycin (linco-spectin) (TI 6.83, SD 7.41 versus TI 2.13, SD 5.65, respectively) and tetracyclines (TI 3.82. SD 12.80 versus TI 3.08, SD 11.83, respectively) were the most commonly used antimicrobials (see Table 2). Linco-spectin use was three times higher in traditional flocks compared to on farm hatched flocks.

**Figure 2:**
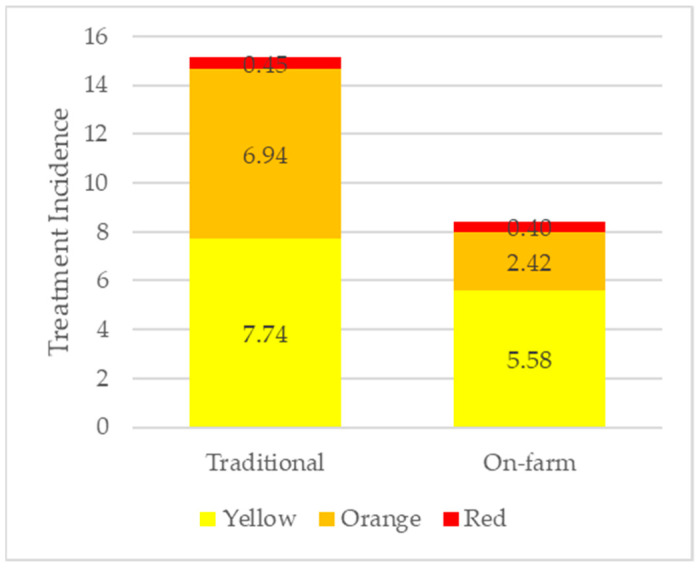
TI of antimicrobials administered to traditional (*n* = 1973) and on-farm (*n* = 118) flocks classified according to the AMCRA formulary (Yellow, Orange, and Red).

Traditional flocks had a higher TI for all AMCRA categories (Figure 2). The largest difference in TI between traditional and on-farm flocks were in the Orange (B) category, where traditional flocks had a TI of 6.94, almost three times greater (*p* < 0.05) than the TI of 2.42 in on-farm flocks.

### 3.2. Indication for AMU

**Figure 3:**
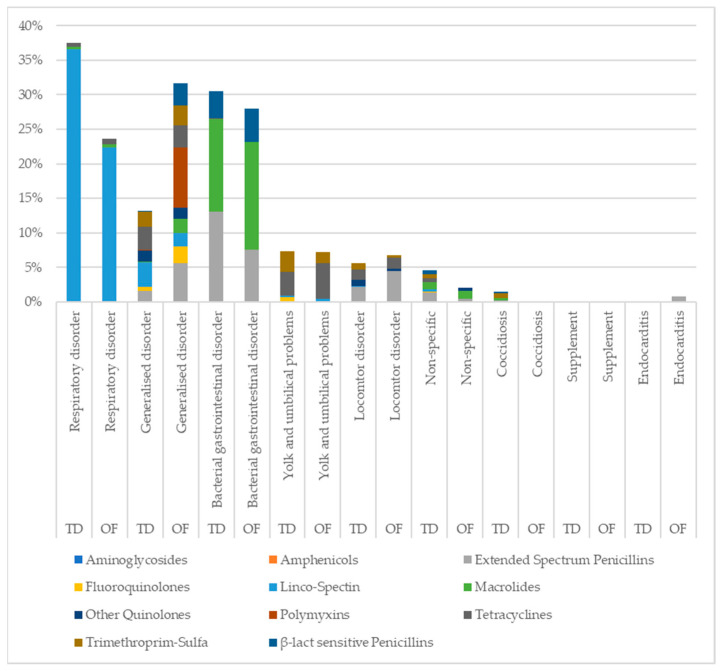
Indication for AMU, including antimicrobial classes, in treated traditional (TD; *n* = 1973) and on-farm (OF; *n* = 118) flocks.

For treated traditional flocks, the most common indication for AMU was respiratory disorders (37.51%, *n* = 1788/4766), followed by bacterial gastrointestinal disorders (30.49%, *n* = 1453/4766). For treated on-farm hatched flocks, the most common indications for AMU were generalised disorders (31.60%, *n* = 79/250) and bacterial gastrointestinal disorders (28.00%, *n* = 70/250) (see Figure 3).

During the first week of the production round, the majority of antimicrobials in both traditional (71.89%, *n* = 1703/2369) and on-farm hatched flocks (61.97%, *n* = 44/71) were administered for the indication of respiratory disorders. In treated traditional flocks, half (56.84%, *n* = 968/1703) of those antimicrobials were administered on day zero. Overall, 22.72% (*n* = 1083/4766) of all antimicrobials administered in treated traditional flocks took place on day zero, compared to 8.00% (*n* = 20/250) in treated on-farm flocks. The treatment incidence for both traditional and on-farm flocks increased during weeks four and five. For traditional flocks, bacterial gastrointestinal disorders were the main indication for treatment during these outbreaks. The increased treatment incidence in on-farm flocks during the same period was largely due to two indications, generalised disorders and bacterial gastrointestinal disorders. A complete overview of the indications is listed in Table 1.

## 4. Discussion

### 4.1. Method of Data Collection and Processing

The quantification of antimicrobial use was based on purchase data of antimicrobial products at the farm level. As the registration of antimicrobial products livestock is mandatory in Belgium, using the purchase data was the most convenient method. While purchase data could be less truthful, as it cannot directly be linked to one specific production round and can, therefore, lead to an overestimation when the purchased antimicrobial product has not been completely administered to the flock, this effect is minimalized in this study, as it followed multiple production rounds at the same farms, and as it is not authorized to keep stocks of antimicrobials in Belgian broiler farms [28]. The DDDvet data, as determined by EVSAC, are based on the recommended dosage as stated in the SPC of the drugs in different European countries. As the recommended dosages can vary for products with the same active substances in different countries, the DDDvet data do not always reflect the actual used dose at flock level and can, therefore, not be used to examine over and underdosing. The DDDvet can, however, be used to quantify antimicrobial use, as has been performed in this research, allowing for comparisons to be made between flocks and farms.

A standardized weight curve was used for the calculation of the ‘kg of animals at risk’. As a large percentage of antimicrobial treatments in broiler production occur early on in the production cycle, using a standard weight of 1 kg would result in an underestimation of the treatment incidence and antimicrobial use.

### 4.2. Husbandry Conditions

Aside from the hatching method, farm and husbandry conditions, which could influence antimicrobial use, such as flock size, flock density, vaccination, and sorting, were similar for both types of flocks included in the study. The conditions for which data were collected were all found to be not statistically significant in their relation to the treatment incidence when inserted in the model used in this study. Furthermore, as 21 of the 28 farms (75%) included in the on-farm sample moved from traditional hatching to on-farm hatching during the sampling period, the similarity in husbandry and farm conditions for the flocks from those farms would be even more so.

In on-farm hatching, unhatched eggs were removed on the day of set up (day 0), whereas the egg shells of hatched eggs remained in the stable and were no longer macroscopically identifiable after around two weeks. Studies have described diverse populations of bacteria on non-disinfected egg shells that contribute to the establishment and succession of the gastrointestinal microbiota [29]. As chicks in on-farm hatching are in contact with the egg shells for a longer period of time in comparison to traditionally hatched chicks, whose contact is limited to the time spent in the hatcher (+/−72 h), it is possible that this longer exposure in on-farm flocks could influence the microbiota or serve as a source for pathogen introduction if pathogens were present on the egg shells. However, all the eggs included in the study, both for traditional and on-farm hatching, were disinfected at the hatchery via ultrasonic treatment before placement in the hatchers. This disinfection method has been shown to effectively reduce the bacteria on egg shells, such as *Escherichia coli* [30]. Thus, the effect of the difference in exposure time to the egg shells should be minimal.

### 4.3. Overall Antimicrobial Use

Farmers using the NestBorn on-farm hatching system used 44% less antimicrobials compared to traditional hatching methods. This lower usage was more pronounced at the start of the production round. Farms using on-farm hatching had 5.6 times higher odds to have a production round without any antimicrobial use compared to traditional farms. Almost half (48.02%, *n* = 109/227) (*p* < 0.001) of the on-farm hatched flocks were antimicrobial free, compared to 12.08% (*n* = 271/2244) of traditional flocks. This was higher than both the 25% of antimicrobial-free cycles observed by Persoons et al. [24] and the 31.2% observed by Kassabova et al. [31]. This supports on-farm hatching to be an effective tool to decrease the need for antimicrobial use in broiler flocks and may increase the number of flocks, which can be raised without any antimicrobials.

### 4.4. Respiratory Disorders and Use of Lincomycin-Spectinomycin

Traditional flocks had a treatment incidence for linco-spectin (TI 6.83, SD 7.41) that was three times higher than that of on-farm hatched flocks (TI 2.13, SD 5.65). Half (53.49%) of all linco-spectin administrations in traditionally hatched flocks took place on day zero, of which the majority (92.99%, *n* = 968/1041) was administered for the indication of respiratory disorders. Overall, the indication of respiratory disorders was the most common indication for treatment on day zero, with 20.31% (*n* = 968/4766) of treatments for respiratory disorders in traditional flocks having occurred on day zero, compared to the 7.20% (*n* = 18/250) of treatments in on-farm hatched flocks. While exposure to high pathogen loads and dust during handling and transportation of day-old chicks [16] in traditional hatching systems could increase their susceptibility to respiratory disorders, the high use of linco-spectin in traditional flocks on day zero is likely the result of routine antimicrobial use, which still occurs in poultry farming [32]. The EU regulation 2019/6, which came into force on 28 January 2022, states that preventive use of antimicrobials is no longer acceptable [8]. As the purchase data used for the current study were obtained before the implementation of the more stringent legislation prohibiting routine or prophylactic use of linco-spectin and other antimicrobials, a similar analysis of antimicrobial use at treatment and flock level using more recent data would be interesting.

### 4.5. Impact of On-Farm Hatching on Intestinal Health

Enteritis is a major concern for the poultry industry, as gastrointestinal disorders result in increased mortality, production loss, and decreased welfare of broilers. Overall, traditionally hatched flocks (28.85%) were treated more for bacterial gastrointestinal disorders than on-farm hatched flocks (19.50%). Flocks from both hatching systems experienced outbreaks of bacterial gastrointestinal disorders during weeks four and five, a typical period for such disorders in broilers [4,33]. An improved gastrointestinal health in on-farm hatched flocks was expected, as direct access to feed and water in on-farm hatching has been shown to positively impact the intestinal health of on-farm hatched flocks compared to that of traditionally hatched flocks. One of the latest studies on the matter, performed by Dieryck et al. [34], showed that serum diamine oxidase levels in hatchery-born chicks were significantly less favourable compared to their on-farm hatched counterparts, suggesting that the intestinal development in the latter took place earlier (diamine oxidase is a marker for intestinal health). These findings of better intestinal health in on-farm hatched broilers form the basis for the expectation of lower antimicrobial use for gastrointestinal disorders, as seen in this study. While feed, a factor that has a major impact on intestinal health and, therefore, the development of bacterial gastrointestinal disorders, was not accounted for in the model, there are no indications to assume that the on farm hatched broilers received different feed compared to the traditional broilers.

### 4.6. Limitations of the Study

As there were 2244 traditional flocks compared to 227 on-farm flocks, the sample was not fully balanced, with the data for traditional flocks being more representative. This was, of course, the result of that fact that on-farm hatching is a relatively new methodology that is not yet largely introduced. Convenience sampling was used to acquire the data used in the current study. While this is time- and cost-effective, it is possible that it creates a level of sampling bias and impacts the representativeness of the farms included in the study [35]. Yet, the used sample in this study is substantially large, which should limit the potential bias. Furthermore, convenience sampling does not allow for an insight into certain aspects, such as the factors influencing the decision of farmers to move to on-farm hatching, which could, in turn, influence the results seen in this study. It is possible that farmers who made the decision to switch to on-farm hatching were, in part, already motivated to use antimicrobials more prudently and were, therefore, incentivized by the company’s claims that flocks hatched using the NestBorn systems require less antimicrobials [36]. On the other hand, it is also possible that farmers were motivated to move to on-farm hatching as a last resort when other measures to lower bacterial outbreaks and the subsequent high antimicrobial use were unsuccessful. Regardless of which scenarios are more prevalent in the farms applying on-farm hatching systems included in this study, it is evident that the motivation of farmers could impact the results of the current study. The impact of farmer motivation and education on antimicrobial use was apparent in a study performed by Caekebeke et al. [37], who found that farmer education on antimicrobial stewardship resulted in a decrease in antimicrobial use by 7%.

## 5. Conclusions

The results of this study indicate that on-farm hatching may contribute substantially to the decrease in antimicrobial use in broilers. With the pressure of AMR, stringent legislation on antimicrobial use and pressure from consumers for a more ethical livestock and poultry production, on-farm hatching systems, which have also been shown to have a positive impact on animal welfare, could play an important role in the future of a more sustainable and ethical broiler production.

## Figures and Tables

**Table 1:** Indications for antimicrobial treatment and included diagnoses per indication category for all traditional and on-farm hatched flocks.

		Traditional		On-Farm	
Indication	Included Diagnoses	Count (*n*)	Percentage (%)	Count (*n*)	Percentage (%)
Locomotor disorder	Arthritis of the knee joint Arthritis of the hock Arthritis of the hock + knee joint Femoral neck necrosis Kinky back Osteomyelitis	268	5.32	17	4.74
Respiratory disorder	Airsacculitis Sinusitis Inflammation of the upper respiratory tract Pneumonia	1788	35.50	59	16.43
Bacterial gastrointestinal disorder	Enteritis Dysbacteriosis Necrotic enteritis Haemorrhagic enteritis	1453	28.85	70	19.50
Coccidiosis	Coccidiosis E. Acervulina Coccidiosis E. Tenella Coccidiosis E. Maxima	71	1.41	0	0.00
Yolk and umbilical disorder	Omphalitis Yolk sac infection Yolk retention	347	6.89	18	5.01
Generalised disorder	Image of bacterial infection Septicaemia Polyserositis Pericarditis	621	12.33	79	22.01
Supplement	Supplement	3	0.06	0	0.00
Endocarditis	Endocarditis	0	0.00	2	0.56
Non-specific	No diagnosis given	215	4.27	5	1.39
	Grand Total	4766	94.62	250	69.64

**Table 2:** Treatment incidence (TI) per antimicrobial class and sum of all treatment incidences for traditional and on-farm flocks.

	Traditional		On-Farm	
Antimicrobial Class	Mean	Standard Deviation	Mean	Standard Deviation
TI Aminoglycosides	0.00 ^a^	0.04	0.00 ^a^	0.00
TI Amphenicols	0.01 ^a^	0.04	0.00 ^a^	0.00
TI Extended Spectrum Penicillins	1.53 ^a^	3.79	1.15 ^a^	4.03
TI Fluoroquinolones	0.24 ^a^	1.74	0.22 ^a^	1.45
TI Lincomycin-spectinomycin	6.83 ^a^	7.41	2.13 ^b^	5.65
TI Macrolides	0.36 ^a^	1.41	0.18 ^a^	0.44
TI Other Quinolones	0.22 ^a^	1.27	0.19 ^a^	1.56
TI Polymyxins	0.01 ^a^	0.26	0.29 ^b^	3.12
TI Tetracyclines	3.82 ^a^	12.80	3.08 ^a^	11.83
TI Trimethroprim-Sulfa	1.66 ^a^	6.52	0.66 ^b^	3.33
TI β-lact sensitive Penicillins	0.45 ^a^	2.80	0.50 ^a^	2.40
Sum of TI	15.11 ^a^	18.01	8.40 ^b^	15.95

Note: Values in the same row with different subscripts are significantly different at *p* < 0.05 in the selected test used (Mann–Whitney test).

## Data Availability

The dataset analysed for this study is available on request from the corresponding author J.G.J. The raw data are not publicly available due to privacy restrictions.
